# Value preference in forensic population: a systematic literature review of delay discounting among those who have committed an offence

**DOI:** 10.3389/fnbeh.2025.1666649

**Published:** 2025-11-19

**Authors:** Ivan Sebalo, Darya Darashkevich, Stela Kostelníková, Johana Voldřichová

**Affiliations:** School of Psychology, University of New York in Prague, Prague, Czechia

**Keywords:** delayed rewards discounting, prisoners, offenders, forensic, decision-making, impulsivity, value preference

## Abstract

**Introduction:**

Delayed rewards discounting describes the tendency to choose a smaller immediate rewards instead of a larger delayed rewards. Considering the central role of impulsivity in models accounting for criminal conduct in general and violent behavior, the relationship between delayed rewards discounting and crime is likely to be present. Thereby extending the reported association with the addictive behavior. However, it is unclear whether it should be treated as a risk or an etiological factor. Consequently, the current literature review aims to summarize the existing empirical research focused on this aspect of impulsive decision-making among those who have offended.

**Methods:**

The review was performed in accordance with the 2021 Preferred Reporting Items for Systematic Reviews and Meta-Analyses (PRISMA) guidelines. The literature search of the Web of Science, PubMed, and PsycINFO databases was conducted in February 2025.

**Results:**

The initial search yielded 1,251 articles. After exclusion of 250 duplicates, 1,001 titles were screened for relevance, leading to 556 abstracts. After reading them, 162 full-text articles were inspected, leaving 25 articles included in the review.

**Conclusion:**

This review demonstrates that although delayed rewards discounting is associated with general criminal conduct, the association with violence specifically is tenuous. Furthermore, several studies point out that influencing serotonergic functioning, behavioral modeling, or future representations have the potential to influence it. However, further detailed research is needed.

## Introduction

Delay discounting refers to the decline in the perceived value of a rewards as the time required to obtain it increases. The longer the waiting time for the rewards the higher is the likelihood for abandoning it in favor of a smaller but immediately available one (Odum, 2011). Delay discounting has been consistently linked with a range of maladaptive behaviors, such as addictions (Weinsztok et al., 2021), dysregulated eating (Stojek and MacKillop, 2017), and attention deficit hyperactivity disorder (Jackson and MacKillop, 2016). Following an impulsive choice that favors a smaller, immediate rewards over a larger, delayed rewards is associated with conduct that can result in potential harm to the individual who makes such a decision.

Furthermore, delay discounting, assessed using computerized tasks where participants expected to receive selected amounts or expected an opponent to receive a negative stimulus, is associated with self-reported sexual coercion, antisocial behavior, and aggression (Bobova et al., 2009; Carrier Emond et al., 2018; West et al., 2022). Similarly, longitudinal research has shown that self-reported impulsivity is related to both life-course and adolescent-limited offending, especially violent behavior (Jolliffe et al., 2017; Farrington and Aguilar-Carceles, 2023). Furthermore, self-reported delayed rewards discounting has been consistently linked with substance use, particularly alcohol (Amlung et al., 2017), which in turn has a robust association with violence (Duke et al., 2018). This places opting for smaller, immediate rewards at the expense of larger, but delayed ones, as a potential risk factor, albeit an indirect one, for criminal conduct.

This aligns with the proposition from the General Aggression Model (Allen et al., 2018). According to it, through evaluative decision-making, aggression is enacted when it is judged to lead to desired values. Likewise, the I^3^ meta-theory of aggression (Finkel and Hall, 2018) places low self-control or a lack of inhibition among the three core factors, which manifest aggressive impulses into conduct. This meta-theory posits that aggression results from evoking proclivity to be aggressive by internal or external stimuli (instigators), which is then boosted or diminished by personal factors (impellance) and is not inhibited by cognitive processes (inhibitors). Indeed, a meta-analysis confirmed that such facets of self-reported impulsivity as negative urgency, sensation seeking, and lack of premeditation were consistently associated with general and physical aggression (Bresin, 2019). Nevertheless, response inhibition, assessed using go/no-go task, exerts different effects on short-lived and continuous aggression among students from the United States (Sebalo et al., 2024). Furthermore, as shown by Madole et al. (2019) behavioral response inhibition and emotional impulsivity exert two separate effects on aggressive behavior.

A potential reason for this is that violent offenders rely on cognitive structures that are different from those of the general population, of the United Kingdom and New Zealand (Polaschek et al., 2009; Sebalo et al., 2023). Thus, the estimated value of various actions for them is likely to be distinct. This would mean that for them, the immediate option of aggressive conduct would not be “the smaller immediate rewards” and being crime-free does not constitute a “larger delayed rewards.” If this is the case, then discounting delayed financial rewards would not translate into a high frequency of violent conduct. Then, delayed rewards discounting stops being an index of impulsivity. Indeed, West et al. (2022) have shown that in the United States, non-offenders high in aggressive or sadistic traits were less likely to discount the infliction of minor harm now in order to inflict more harm in the future.

Similarly, Åkerlund et al. (2016) reported that males who, at the age of 13, discounted larger but delayed hypothetical rewards in a task in favor of a smaller but immediate one, had a higher likelihood of obtaining a criminal conviction by the age of 31 for any crime, including property and violent ones, as compared to those who did not. However, the association weakened when the total number of crimes was ken into account. Likewise, Lee et al. (2017) have shown that although there is a correlation between delay discounting in a hypothetical task and self-reported engagement in any criminal activity, the interaction between these two variables is not straightforward. While the rate of discounting larger delayed rewards was associated with property crime a year later, it was not associated with property crime 2 years later. At the same time, for violent crime, the relationship was reversed. Self-reported violent behavior was associated with higher rates of choosing smaller and immediate rewards a year later, but not the other way around. These results highlight two aspects. First, delayed rewards discounting appears to be a risk factor for engaging in criminal conduct. Second, rather than being a trait characteristic, it is a preference, the rate of exercising, which can be affected by other behaviors.

Considering the high prevalence of impulsivity in forensic populations and its association with aggression (Tonnaer et al., 2016), steep delay discounting can be expected to be prevalent in this population. After all, despite certain reductionism, Gottfredson and Hirschi’s (1990) the general theory of crime posits a deficiency in self-control as the core reason for crime (Burt, 2020). However, a potential complication of the relationship between delayed reward discounting and criminal conduct arises from the realization that such conduct can be perceived as rewarding for those who engage in it. Moreover, given the well-founded argument for reconceptualizing impulsivity as a construct, a systematic investigation focused on this particular aspect of impulsiveness among those who have committed a crime is warranted.

Consequently, the current study aims to summarize the existing research studying delayed rewards discounting among adults who have committed any criminal offense. This allowed to verify whether choosing smaller immediate rewards over larger but delayed ones can be considered a precursor for criminal conduct of any magnitude or nature. When the included studies specified the violent or non-violent nature of the offense, the difference in patterns was captured. Furthermore, it provides a description for choice preferences and associated constructs specifically among those who were found legally guilty of an offense. The choice to focus on forensic^1^ rather than on the general population was based on the uniqueness of the former. Understanding the etiology and risk factors of criminal conduct necessitates studies involving those whose engagement in it was proven beyond a reasonable doubt.

## Materials and methods

The literature review was conducted in accordance with PRISMA guidelines (Page et al., 2021). It was conducted the February 2025, when Wed of Science, PsychINFO and PubMed were searched using the following terms: Psychopathy OR Offend* OR Convict OR Criminal OR prisoners OR felons OR convicts OR inmates OR Antisocial” AND intertemporal choice OR delay discounting OR delayed rewards discounting OR temporal discounting OR delayed gratification OR delayed gratification OR impulsive choice OR impulsive decision making OR intertemporal decision making OR intertemporal choice OR discounting.

Studies were eligible for inclusion if they met the following criteria: were published in English; had a quantitative design; were published in a peer-reviewed journal; assessed delayed rewards discounting and criminal conduct after the age of 18.

Given the study’s aim, the review included only studies that had samples drawn from the forensic population, which is distinct from the general population. The primary criterion was the presence of a criminal conviction. Studies that relied on self-reports of behaviors considered to be criminal were not included, because self-reports do not equate with a legally determined sentence. For similar reasons, although the diagnosis of Antisocial Personality Disorder (ASPD) and psychopathy contains criminal versatility as one of the symptoms, unless the presence of criminal conviction among participants was explicitly stated, the study was excluded.

**FIGURE 1:**
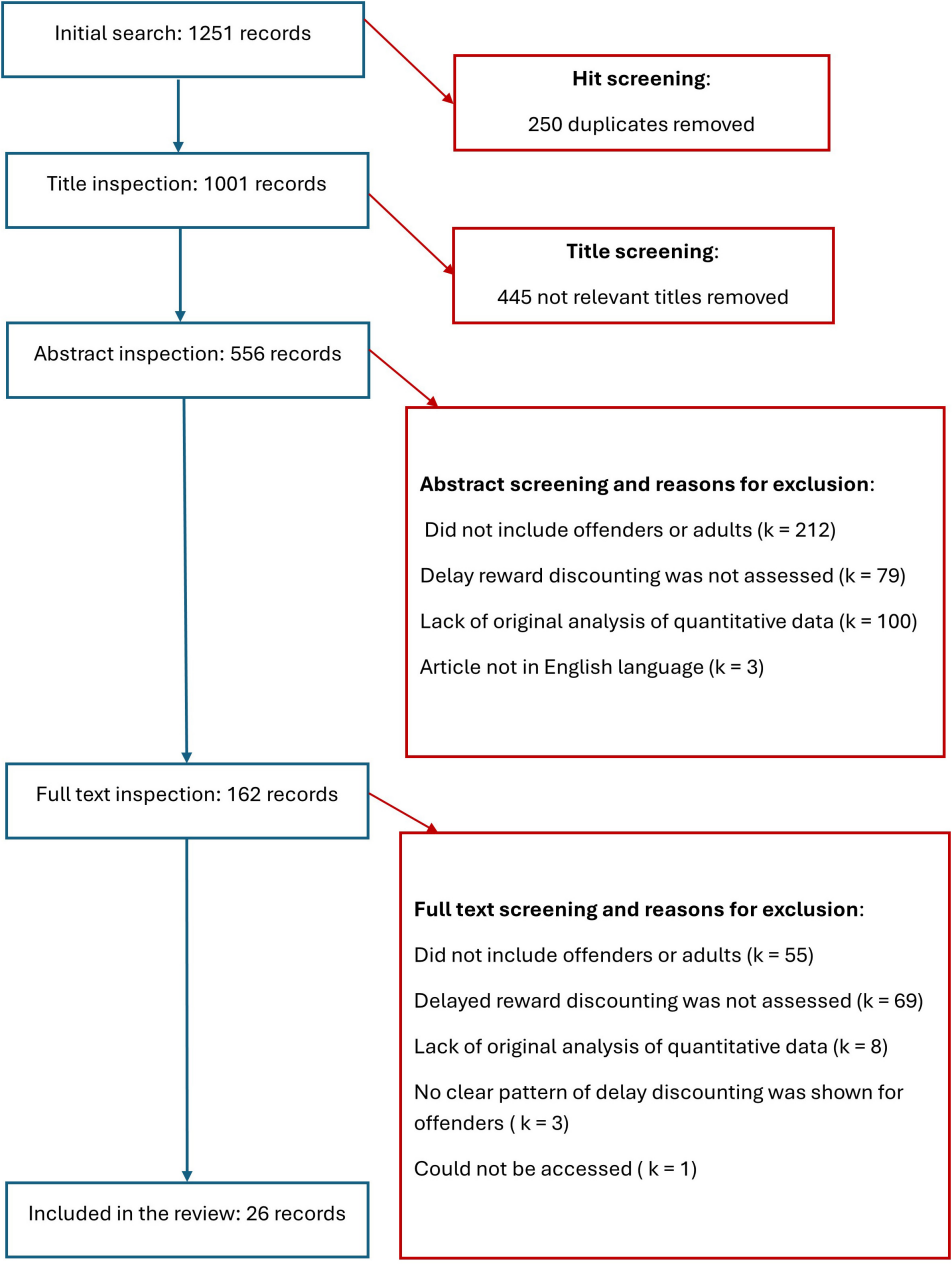
Flowchart of study inclusion.

The initial search yielded 1,251 articles, with 250 duplicates. The remaining 1,001 titles were inspected for relevance. After removing 445 non-relevant titles, 556 abstracts were inspected. Of these, 162 were found to be relevant. Following a full-text inspection of the articles, with particular attention to the assessment of criminal conduct and delayed rewards discounting, 26 articles were found suitable for inclusion in the review. Four researchers reviewed the titles, abstracts and full texts, and performed a quality check. One study was identified as having poor quality; however, it was kept in the review to keep transparency and maintain completeness of the results. The details of the excluded studies are presented in Figure 1.

### Study characteristics

The majority of studies were conducted in the United States of America (*n* = 13), followed by the United Kingdom (*n* = 5), and Australia (*n* = 2). Countries with only one study included China, the Netherlands, and New Zealand. There was also one cross-cultural study that was conducted in Italy and Poland. The majority of studies were cross-sectional quasi-experimental (*n* = 8), as they were conducted at one point in time but compared different groups. There was almost equal amount of purely cross-sectional (*n* = 7) and experimental (*n* = 6) studies, and two longitudinal ones. More detailed information on the included studies is provided in Table 1.

**TABLE 1:** Summaries of included studies.

Referenes	Country	Research design	Sample	Measure of delayed rewards discounting	Summary of findings	Quality check
Arantes et al., 2013	New Zealand	Cross-sectional / quasi-experimental	133 participants, 63 incarcerated (36 males and 27 females), 70 community (28 males and 42 females)	16 items relating to delayed monetary rewards.	Offenders demonstrated significantly higher discounting rates than non-offenders, as they prioritized short, immediate, smaller rewards	2
Brennan et al., 2010	United Kingdom	Cross-sectional / quasi-experimental	40 male prisoners, 15 weapon violent, 10 non-weapon violent, 15 non-violent	Choice task adapted from Stewart et al. (2003).	Weapon-violent offenders had the lowest delay discounting rates of all groups. However, mean differences between the respective groups were small and not significant.	2
Brown and Gutsch, 1985	United States of America	Cross-sectional	53 male prisoners: 20 diagnosed with secondary and 17 diagnosed with primary psychopathy	The delay of gratification questionnaire was inspired by Mischel and Grusec (1967)	Those with primary psychopathy chose the longest delay significantly more than those with secondary psychopathy and those without this diagnosis	1
Cherek and Lane, 1999b	United States of America	Quasi-Experimental	30 females on parole, 10 with a history of violent and 20 with a history of non-violent crime	A modified version of the self-control paradigm introduced by Mazur (1987) was used to measure impulsive behavior.	Participants with a history of violent crime chose the immediate and smaller rewards option significantly more than those with a history of non-violent crime. There was a significant negative correlation between choosing immediate and smaller rewards and aggression for females with a history of non-violent crime, but it was not significant for those with a history of violent crimes. However, there was a significant positive correlation between choosing immediate and smaller rewards and aggression among males with a history of violent crime, but not for males with a history of non-violent crime.	1
Cherek and Lane, 2000	United States of America	Quasi-Experimental	10 males on parole: 5 with a history of conduct disorder and 5 without	A modified version of the self-control paradigm introduced by Mazur (1987) was used to measure impulsive behavior.	There was a significant decrease in choosing an immediate but smaller reward following administration of d,l-fenfluramine, but only among participants who were diagnosed with conduct disorder	1
Cherek and Lane, 1999a	United States of America	Experimental	10 males with a diagnosis of conduct disorder and antisocial personality disorder on parole	A modified version of the self-control paradigm introduced by Mazur (1987) was used to measure impulsive behavior.	There was a significant dose-dependent decrease in choosing immediate but smaller rewards following administration of d,l-fenfluramine.	1
Cherek and Lane, 2001	United States of America	Quasi-Experimental	10 males on parole: 5 with a history of conduct disorder and 5 without	A modified version of the self-control paradigm introduced by Mazur (1987) was used to measure impulsive behavior.	Rates of choosing the delayed option with higher rewards decreased, not not-significantly following D-fenfluramine administration.	1
Cherek et al., 2002	United States of America	Experimental	12 males with a history of conduct disorder on parole	A modified version of the self-control paradigm introduced by Mazur (1987) was used to measure impulsive behavior.	There was a significant decrease in choosing the immediate option with a smaller rewards following a chronic administration of paroxetine	1
Cherek et al., 1997	United States of America	Quasi-Experimental	30 males on parole, 9 with a history of violent crime and 21 with a history of non-violent crime	A modified version of the self-control paradigm introduced by Mazur (1987) was used to measure impulsive behavior.	Participants with a history of violent crime chose the immediate and smaller rewards option significantly more than those with a history of non-violent crime	1
De Brito et al., 2013	United Kingdom	Cross-sectional / quasi-experimental	45 male offenders: 17 diagnosed with ASPD and psychopathy, 28 diagnosed with ASPD but not psychopathy, 21 males without a criminal conviction	Cambridge Gamble task	There was no significant difference in the rates of choosing immediate and smaller rewards between those with ASPD and psychopathy diagnoses, those with only ASPD diagnoses and those without a criminal conviction.	2
Hanoch et al., 2013	United Kingdom	Cross-sectional / quasi-experimental	89 male offenders in prison, or 16 weeks after their release, and 106 participants without a criminal conviction	The Monetary choice questionnaire (MCQ) (Kirby and Maraković, 1996)	Released prisoners, but not those who are currently in prison, were more likely to choose immediate and smaller rewards than those without a criminal conviction, non-offenders, particularly when delayed rewards were medium or large	2
Hosking et al., 2017	USA	Cross – sectional	49 male offenders in prison.	A computer-based procedure using hypothetical monetary choices	Delay discounting rate was not significantly associated with the number of committed crimes	2
Huddy et al., 2017	United Kingdom	Cross-sectional	72 male prisoners	The Monetary choice questionnaire (MCQ) (Kirby and Maraković, 1996)	Discounting delayed rewards was significantly associated with opiate use, but not crack or cocaine use or personality disorder symptoms	1
Iozzino et al., 2022	Italy and Poland	Cross-sectional / quasi-experimental	115 patients with schizophrenia spectrum disorders, 74 were from forensic services and had a history of violent conduct, 41 had no such history and were from general psychiatric hospitals	computerized Cambridge Gambling task	There was no significant difference in the rates of choosing immediate and smaller rewards between those from forensic and non-forensic services	2
Jones et al., 2015	Australia	Cross-sectional	68 men and 12 women with one or more convictions in the last 5 years. 101 university students without a conviction	A computer-based procedure using hypothetical monetary choices	Those without a criminal conviction were less likely to discount delayed and larger rewards than those with a criminal conviction.	2
Jones et al., 2013	Australia	Experimental	204 attendees of the drug court, 96 were under intense supervision, and 108 received supervision as usual.	Nine large magnitude rewards items from the Monetary Choice Questionnaire (MCQ) (Kirby and Maraković, 1996)	Those who more often chose delayed and larger rewards and were under intense supervision had significantly lower odds of substance use than those under normal levels of supervision. Those who chose immediate and smaller rewards under either amount of supervision had a similar likelihood of substance use as those who opted for larger delayed rewards under normal levels of supervision	1
Lijffijt et al., 2017	United States of America	Cross-sectional / quasi-experimental	38 males diagnosed with ASPD and with a criminal conviction, and 18 community participants with no diagnoses or criminal conviction	Two-Choice Impulsivity Paradigm (TCIP) Dougherty et al., 2003, based on a modified version of the self-control paradigm introduced by Mazur (1987), was used to measure impulsive behavior.	Those with a criminal conviction and ASPD diagnoses did not differ in delayed rewards discounting from those without either.	2
Newman et al., 1992	United States of America	Experimenta	158 male prisoners, 73 with psychopathy, 85 without	The delay of gratification task	Those diagnosed with psychopathy and low levels of anxiety opted for immediate and smaller rewards less frequently than those without psychopathy but similar levels of anxiety.	0
Peng et al., 2022	China	Cross-sectional / quasi-experimental	518 offenders(386 male, 132 female) and 636 community participants (452 male and 184 female)	The Monetary choice questionnaire (MCQ) (Kirby and Maraković, 1996)	The delayed rewards discounting rate was significantly associated with the likelihood of offending.	2
Pietras et al. (2003)	United States of America	Experimental	11 males with a criminal conviction	A modified version of the self-control paradigm introduced by Mazur (1987) was used to measure impulsive behavior.	Administration of methylphenidate resulted in a decrease in choosing immediate and smaller rewards	1
Piquero et al., 2018	United Kingdom	Longitudinal	411 male members of the Cambridge Study in Delinquent Development (CSDD)	Three questions: At age 18, “I would rather have £10 now than £20 in a year”; at age 32, they were asked, “I would rather have £50 now than £100 in a year” and at age 48, they were asked, “I would rather have £50 now than £100 in a year”	Even when accounting for established environmental and individual risk factors, delayed rewards discounting was a significant predictor of the number of convictions.	2
Rieser et al., 2019	Netherlands	Longitudinal	215 males with substance use disorder on parole	A computerized version of the Delay Discounting Task by Wittmann et al. (2007)	The delay discounting rate was not significantly associated with violent or property crimes.	2
Sloan et al., 2014	United States of America	Cross-sectional	1,567, with 40 reporting to be arrested	Four questions: “Would you rather win $1000 now or $1500 a year from now?” “Would you rather win $20 now or $30 a year from now?” “Would you rather lose $1000 now or $1500 a year from now?” “Would you rather lose $20 now or $30 a year from now?”	Drinking drivers tend to show greater impulsivity but not greater responsiveness	2
Stumphauzer, 1972	United States of America	Experimental	40 male prisoners	A pool of choices between immediate and delayed but more valuable rewards	Witnessing someone opt for delayed and larger rewards significantly increased the frequency of delayed choices for up to 1 month.	1
Torres et al., 2023	United States of America	Cross-sectional / quasi-experimental	35 participants with CUD, 19 with a history of incarceration(18 males and 1 female), 16 with no history of incarceration (14 males and 2 females)	Personalized delay discounting task, where participants decided between $10 available “today” or a different amount, available at some delay.	When non-systematic responders were excluded, those with a history of incarceration opted for immediate and smaller rewards more often than those without it. Imagining oneself in future contexts led to a significant reduction in delay discounting among both those with and without a history of incarceration.	2
Varghese et al., 2014	United States of America	Cross-sectional	146 male ex-prisoners within 5 months of release.	The Monetary choice questionnaire (MCQ) (Kirby and Maraković, 1996)	Those with more incarcerations were significantly more likely to choose immediate and smaller rewards rather than delayed and larger ones. Criminal thinking style was significantly correlated with temporal discounting. The temporal discounting is associated with reactive criminal thinking but not with proactive one.	1

^1^In this article forensic population refers to those who have committed a criminal offense regardless of the presence of absence of a psychiatric diagnoses and the type of crime.

^2^V = A/(1−*k*D), where V is the perceived values of an outcome; A is an absolute value; D is delay; and k is the discount rate that determines the slope of the discounting function.

The quality of the included studies was assessed using Checklists for Analytical Cross-Sectional Studies, Cohort Studies, Quasi-Experimental Designs, and Randomized Control Studies from the Joanna Briggs Institute (JBI) at the University of Adelaide (Moola et al., 2020). For each item of the checklist, the rating was rated as either present, absent or not applicable, and then a sum score was calculated. One author (IS) rated the included studies separately from the three authors. Two disagreements were resolved in a discussion. There was almost an equal number of studies with excellent quality (*n* = 13), defined as having a maximum score or of 1 below, and good quality (*n* = 12), defined as having a score above the median plus 1 and below excellent. One study was of poor quality, but it was retained. As noted in the previous section, studies were only included if they captured criminal conviction, which in most cases was captured through examination of participants’ criminal records. There were only two studies where participants self-reported their incarceration or arrest.

### Delayed rewards discounting measurements

All of the included studies assessed delayed rewards discounting by presenting participants with two options: one associated with a smaller but immediate gain and another with a larger rewards, but after a delay. However, the specific paradigms and instruments were different.

An amended version of the self-control task from Mazur (1987), titled the *impulsivity paradigm (IMP)* was the most widely used (*n* = 8). During it, two letters appeared on the screen. As soon as one is selected, it remains on the screen and, after a delay, starts to flash. Pressing on it again would yield a set rewards. One of the options started to flash after 5 seconds and yielded 5 cents, while the other one began to flash after 15 s and yielded 15 cents. Participants were paid based on the amount they had earned during the sessions.

A particular variation of the IMP was used in one study (Newman et al., 1992). Participants were presented with two buttons on the screen, where participants could choose either one. The rewards were always 5 cents (represented by a poker chip); however, the consistency and delay differed depending on the condition. In rewards, only the first group button was rewarded 40% of the time and could be pressed immediately, while the other button was rewarded 80% of the presses and could be pressed 10 s after the start of the trial. In the equal condition, the frequency of rewards was the same, but both options could be chosen 10 s after the start of the trial. Lastly, in the rewards and punishment group, one option was rewarded 90% of the presses and penalized (a subtraction of 5 cents) 10% of the trials, and could be pressed 10 s after the start. Meanwhile, the other option could be chosen 5 s after the start, but was rewarded in 70% of trials and punished in 30%.

The full version of the *Monetary choice questionnaire (MCQ)* from Kirby and Maraković (1996) was the second most consistently used (*n* = 4) measure. MCQ is a 27-item questionnaire where participants must choose one of two monetary options. Original MCQ includes three groups of rewards values: small ($30–$35), medium ($55–$65), and large ($70–$85). However, the currency and amount were adjusted based on the country where the study took place and the time it was conducted. For instance, “Would you prefer £27 today or £28 in 117 days?” (Hanoch et al., 2013). One choice is smaller but immediate, and another choice is larger but delayed. The magnitude of the rewards and the delay, ranging from 7 to 189 days, differ between trials. Using Mazur’s (1987) discounting equation,^2^ the rate of discounting can be calculated.

Jones et al. (2013) used only *nine, larger rewards value questions* from MCQ. Meanwhile, Jones et al. (2015) used a *computerized version* inspired by it. In this task, the delayed gain or loss was always 10$ or 100$. There were also studies (*n* = 5) that used similarly phrased *questions*, but the number of questions and the magnitudes of the rewards were different.

Additionally, a *16-item questionnaire* was used by Arantes et al. (2013). In it, participants were asked to imagine they had won a lottery and could choose either the money now or a larger sum after a certain delay. The immediate rewards and delays also varied, with immediate rewards being $500, $1,000, $2,000, and $4,000 and delay options being 1, 2, 4, and 8 years. The main difference, though, was imagining a lottery win and asking to write the sum that would make both options equally attractive.

Rieser et al. (2019) used a *computerized version of the delay discounting task* (Wittmann et al., 2007), which operates on the same principles of varying magnitude of awards, with immediate rewards ranging from 0 to 476 euros and delayed rewards ranging from 476 to 524 euros and delays being either 5, 30, 180, 365, 1,095, or 3,650 days. It consists of 48 trials, and the outcome measure is the area under the discounting curve.

A slightly different version of *the computerized delay discounting task was used by* Hosking et al. (2017). It also included 48 trials, but both short- and long-term rewards varied in time and magnitude. Short-term rewards ranged between 5$ and 40$ and could be immediate, within 2 or within 4 weeks. Long-term rewards were dependent on the short-term ones and constituted 1%, 3%, 5%, 10%, 15%, 25%, 35%, or 50% increase. Their delay ranged from 2 to 4 weeks from the short-term one.

*The Cambridge Gambling Task* was used in two studies. During it, participants saw 10 boxes on a screen and needed to guess behind which one of them a token was hidden and place a bet, in the form of a percentage of the 100 points they had for each trial. The magnitude of the bet was presented either in descending or ascending order. Matching them allowed to determine those who will wait for a high bet in either circumstance and those who opt for the immediate option at the cost of a delayed one but promising higher benefit.

Lastly, one study employed a *unique measure*. Brown and Gutsch (1985) presented participants with choices concerning objects available in prison, specifically cigarettes, Coca-Cola, pens, and chocolate bars. Participants could choose either the smallest amount immediately or an increasing amount after 6 h, 2 days or 1 week. Participants were also informed that they would be given one of their chosen options selected at random.

## Results

### Group comparison

A third of the studies (*n* = 9) compared the patterns of delayed rewards discounting in offenders and those who had not committed any crime. The majority of them (*n* = 7) showed a clear and significant difference between these two populations. Those who have committed a crime showed higher discounting rates as they consistently preferred smaller short-term rewards to larger but delayed ones (Arantes et al., 2013; Hanoch et al., 2013; Jones et al., 2015; Peng et al., 2022; Torres et al., 2023). Neither of these studies has differentiated between specific types of offense, suggesting that committing one has a relationship with choosing immediate rewards, even if it is smaller than a delayed one. Although Sloan et al. (2014) focused specifically on comparing drunk drivers with other drinkers, the same patterns emerged.

Arantes et al. (2013) specify this preference as “on average, non-offenders reported that $2,520 delayed 1 year was equal in subjective value to $1,000 now, whereas the corresponding indifference point for offenders was $8,183” (p.247). Their study included both males and females from medium secure prisons and community participants and reported a medium to large effect size of the difference. Meanwhile, Peng et al. (2022) demonstrate that even when covariates, such as relative deprivation, sensitivity to rewards and punishment, and decision-making styles are present, delayed rewards discounting still has a significant relationship with unspecified offending. Hanoch et al. (2013) clarified this relationship further. Both those who are or were imprisoned, primarily for drug-related and violent offenses, showed steeper delayed rewards discounting than community participants. However, despite the significant differences, the effect size was small. Likewise, Jones et al. (2015) showed that those with a criminal history, the majority with convictions for theft and burglary, discount delayed rewards more steeply than those without it. Although the study focused on those from the drug court, discounting delayed rewards was not associated with illicit drug use (i.e., failure to comply with programme requirements). However, ex-prisoners discounted the delayed rewards with higher frequency than those currently in prison or community participants. This lends to a supposition that delayed rewards discounting is a habit that develops with experience rather than a stable trait. Indeed, Torres et al. (2023) show that imagining oneself in the future can reduce discounting among veterans with a history of incarceration, for any crime, as well as without. The authors did not report effect sizes, though.

However, there were also studies reporting non-significant differences between those with a criminal conviction, primarily for violent offenses, and those without (De Brito et al., 2013; Lijffijt et al., 2017). One potential reason for these deviations is the task chosen to assess delayed rewards discounting. Rather than asking participants to choose between a certain amount of money now vs. a differing amount later, Lijffijt et al. (2017) the amended version of the self-control task, Mazur (1987) titled the Two Choice Impulsivity Paradigm (TCIP), requires inhibiting an immediate response rather than expressing one’s preference, as was done by aforementioned studies. Meanwhile, De Brito et al. (2013) used gambling task where participants needed to wait before the bet increased in value, which again requires response inhibition rather than just preference expression. Given that these types of tasks assess the behavioral expression of delayed rewards discounting, the aforementioned difference may apply to the cognitions rather than conduct.

### Offenders only

Slightly more than half of the studies included in this review (*n* = 16) focused exclusively on offenders when investigating the constructs associated with delayed rewards discounting. Their results showed potential associations with psychopathy (Brown and Gutsch, 1985; Newman et al., 1992; Hosking et al., 2017), stimulants (Pietras et al., 2003), incarcerations (Varghese et al., 2014), serotonin (Cherek and Lane, 1999a,^2000^, ^2001^; Cherek et al., 2002), and tenuous associations with violence (Cherek et al., 1997; Cherek and Lane, 1999b; Brennan et al., 2010), modeling (Stumphauzer, 1972) and substance use (Jones et al., 2013; Huddy et al., 2017). Although most of these relationships are based on a small number of studies, in some cases, even just one, they can serve as a potential guideline.

For example, Brown and Gutsch (1985) reported that male offenders, in a medium security prison, with primary psychopathy chose large rewards with lower delays when compared to those with secondary psychopathy or no such diagnosis. However, Newman et al. (1992) showed that male offenders, in a minimum security prison, diagnosed with psychopathy and low levels of anxiety discounted delayed rewards less frequently when compared to those without psychopathy but with similarly low anxiety. However, this difference was evident only when expressed preferences led to actual rewards. These two studies suggest that although psychopathy can be relevant to the pattern of delayed rewards discounting, it is unlikely to be the sole determinant. However, it is important to highlight that Newman et al.’s (1992) study was the only study of poor quality and that both studies did not report effect sizes. A more recent study has also reported no direct association between delay discounting rate and psychopathy symptoms or criminal convictions, among those currently in medium secure prison (Hosking et al., 2017). However, they did demonstrate the presence of weaker medial cortico-striatal functional connectivity among those with high psychopathy traits during delay discounting tasks, which was associated with criminal convictions.

Likewise, when it comes to ivolence, the results are not consistent. Brennan et al. (2010) reported that despite lower delay discounting rates among offenders with a history of weapon-involved violent crime as compared to those with a history of violent crime without a weapon or non-violent crime at all, these differences were not significant. Although the authors suggest that small sample size prevented them from detecting effects, prior studies with even smaller samples found the relationship to be significant for both male and female offenders on parole, but did not specify the effect size (Cherek et al., 1997; Cherek and Lane, 1999b). Specifically, violent parolees had higher delay discounting rates than non-violent ones. However, a significant moderate negative correlation was reported between delay discounting and aggression for non-violent females, but not for violent ones.

Meanwhile, for males, it was reversed. There was a significant strong positive correlation between delay discounting and aggression among violent males, but not among non-violent males. Taken together, these findings again point to the possible relevance of preferring short-term rewards over delayed ones in understanding violent behavior, but undermine its position as a core determinant. Nevertheless, the relationship with criminal offenses in general, described in the previous section, appears to extend to the number of criminal convictions, of varying severity, and reactive criminal thinking, assessed in a sample of prisoners released primarily from minimum-security prisons (Varghese et al., 2014).

The relationship with serotonin, however, was slightly more straightforward. Cherek and Lane, 1999a,^2000^) reported an apparent decrease in delay discounting following d,l-fenfluramine (a 5-HT releasing agent) administration among participants with a history of conduct disorder who were on parole, after having an unspecified conviction. However, the lack of sizes precludes the specification of the magnitude. Furthermore, in a subsequent study, despite the presence of a trend, this association was not significant (Cherek and Lane, 2001). Attributing the lack of significance to a smaller sample size, Cherek et al. (2002) showed that administration of paroxetine (a 5-HT reuptake inhibitor) also decreases, albeit to an unspecified magnitude, delay discounting, particularly at the last day of treatment, which took place 2 weeks after the baseline. Consequently, the positioning of serotonin as an active agent in determining the preference for short-term or delayed rewards appears justified. The results showing that serotonin plays an important role in facilitating the ability to prefer larger rewards later at the expense of a smaller rewards immediately align with Miyazaki et al.’s (2012) argument, based primarily on animal studies. Furthermore, they match the suggestion that serotonin affects impulsive aggression through its impact on threat reactivity and harm aversion (da Cunha-Bang and Knudsen, 2021). Administration of methylphenidate also resulted in unspecified decreased delay discounting among males who committed any crime and without a history of ADHD diagnosis or treatment (Pietras et al., 2003). This would also implicate the functioning of dopamine and norepinephrine.

Another common correlate of rewards discounting, namely drug use, was investigated only in two studies that included only participants with a criminal conviction. Huddy et al. (2017) showed that among those in high secure prisons, high delay discounting had a small bivariate association with opiate use, but in a regression model, it did not predict crack or cocaine use or personality problems. Meanwhile, Jones et al. (2013) have shown that low discounting decreases the chances of drug use more efficiently when there is intense supervision of those who have committed any offense than when there is supervision as usual. However, high discounters did not differ from low discounters when the amount of supervision was normal. Furthermore, the effect of intense supervision was varied as the odds ratio ranged from 0.36 to 0.99. Taken together, these results, again, do not show a uniform and robust relationship between substance use and delayed rewards discounting among prisoners.

Lastly, there was one dated study, which had acceptable quality and was among the two assessing a non-pharmacological attempt to influence delayed rewards discounting. Stumphauzer (1972) have shown that exposure to someone else choosing a delayed rewards leads to a significant increase of unspecified magnitude in delay with effect lasting for up to a month, among those currently in a medium secure prison. This highlights a promising technique for counteracting delayed rewards discounting and highlights the need to investigate the difference between capacity and motivation. The inability to control the preference for immediate rewards over larger ones later is not the same as a disinterest in evaluating both options and making a reasoned choice. More replications and further studies are needed, though.

### Longitudinal

There were also two longitudinal studies examining delayed rewards discounting and offending behavior. However, both have opposing results. Rieser et al. (2019) followed 213 male offenders on parole with SUD diagnoses for 14.4 months on average. Their results showed no association between delay discounting rates at the baseline and substance use at the follow-up. Likewise, they have reported that delayed rewards discounting is a non-significant predictor of property crimes, violent crimes, or any crime. However, Piquero et al. (2018) followed the convictions of 411 males from the Cambridge Study in Delinquent Development (CSDD) from the age of 18 until 56, and delayed rewards discounting was assessed at the ages of 18, 32, and 48. They have found delayed rewards discounting rate to be a positive predictor of small magnitude for the number of convictions throughout adulthood, even when accounting for other individual and environmental variables (e.g., IQ, extraversion, low family income). Importantly, once other factors were controlled for the effect of delay discounting became more consistent as the low boundary of incidence rate ratio changed from 1.001 to 1.018. Considering the length and scope of the latter study, which included a wide range of offenders, it appears that a preference for immediate but smaller rewards over delayed but larger ones is associated with criminal conduct. However, while the former study assessed delayed rewards discounting using an extensive task, the latter only used three questions.

## Discussion

This literature review aimed to summarize the empirical evidence about the delayed rewards discounting among adults with criminal convictions. All but one of the included studies were of good or excellent quality based on the evaluation forms from the Joanna Briggs Institute (JBI) at the University of Adelaide (Moola et al., 2020). They also included at least partially participants with a recorded criminal offense and assessed delayed rewards discounting by presenting two mutually exclusive options. Taken together, the results highlight three key aspects. First, adults who have committed at least one crime tend to opt for smaller, immediate rewards at the expense of larger, delayed ones. However, steeper delayed rewards discounting appears to be a predisposing rather than a sufficient factor of small magnitude in males for future offending, particularly over long periods. Second, although preference for smaller gains right now rather than larger ones after some time is associated with serotonergic functioning, the association with psychopathy, substance use, and violence or criminal thinking needs to be tested further, as the current results are conflicting and come from relatively small samples. Lastly, two studies (Stumphauzer, 1972; Torres et al., 2023) have shown that delayed rewards discounting among offenders can be reduced, suggesting the malleability of this aspect of impulsivity.

The association between delay rewards discounting and registered criminal offenses among adults (Arantes et al., 2013; Hanoch et al., 2013; Varghese et al., 2014; Jones et al., 2015; Peng et al., 2022; Torres et al., 2023) agrees with findings based on self-reported antisocial or offending behavior (Bobova et al., 2009; Carrier Emond et al., 2018; West et al., 2022). It also appears to lend support to the central positioning of cognitive structures and processing in the General Aggression Model (Allen et al., 2018) and I^3^ meta-theory (Finkel and Hall, 2018). However, considering weak evidence for the link between delayed rewards discounting and violent behavior (Cherek et al., 1997; Cherek and Lane, 1999b; Brennan et al., 2010), this support should be considered partial. A potential explanation for this inconsistency may lie in understanding the rewards magnitude and its nature. The hidden premise behind the suggested relationship of delayed rewards discounting and aggression is that perpetrating aggression yields a smaller but immediate rewards (e.g., maintaining status) that is chosen instead of a larger rewards of a crime-free life and maintenance of the social contract. However, these magnitudes are not necessarily shared by an offender. Indeed, Varghese et al. (2014) has shown that discounting of delayed monetary rewards was associated with reactive but not proactive criminal thinking. Furthermore, West et al. (2022) have shown that non-offenders with antagonistic traits are more likely to choose the delayed option if it promises larger harm. In other words, it is possible that for offenders, the tendency to discount larger but delayed financial rewards does not necessarily transfer to the tendency to discount larger but delayed intrinsic rewards that they can achieve through aggressive conduct.

Nevertheless, these findings clearly show that delayed financial rewards discounting is associated with criminal conduct, albeit with a small to moderate strength. The results from one of the longitudinal studies strengthen this relationship (Piquero et al., 2018). A possible inference from this is that for offenders, crime in general is more related to financial rewards than violence. This would support the proposition of Gottfredson and Hirschi’s (1990) general theory of crime. Furthermore, this aligns with Lee et al.’s (2017) findings demonstrating a relationship between delayed rewards discounting and self-reported property crimes rather than violent crimes. Nevertheless, the proposed separation of crime in general and violence does not fully agree with the findings of Åkerlund et al. (2016). They have reported that delayed rewards discounting during adolescence was associated with at least one violent crime within the next 28 years. Concerning Varghese et al. (2014) findings, it is possible that the violent crime was predominantly reactive rather than proactive. Another possibility for the differences is that Åkerlund et al. (2016) assessed delayed rewards discounting at the age of 13, possibly assessing general impulsivity rather than choice preference.

Nevertheless, the other longitudinal study included in the review did not find any association between delayed rewards discounting and either general or violent or property crime within 12 months (Rieser et al., 2019). The immediate reasons for this discrepancy appear to be the focus only on offenders with SUD diagnoses and a relatively short duration. Still, the lack of association places delayed rewards discounting into the risk factors category rather than the causes.

However, even as a risk factor, delayed rewards discounting is not the optimal strategy for social or financial situations, which raises the question of whether it can be influenced. A set of studies demonstrated that an increase in serotonin, potentially through an increase in inhibitions, can increase the preference for larger delayed rewards (Cherek and Lane, 1999a, 2000). Moreover, there was promising, albeit preliminary, evidence for the effectiveness of non-pharmacological interventions. Stumphauzer (1972) demonstrated that simply witnessing a person opting for the delayed option can increase similar choices among adult prisoners. Meanwhile, Torres et al. (2023) demonstrated that personalizing the date of the delayed rewards can facilitate the refusal of the immediate but smaller gain. Although replications and further investigation are necessary to verify the utility of such interventions, the changes itself is noteworthy. It suggests that delayed rewards discounting can change depending on the environment and the individual’s cognitive structures. This, in turn, lends support to the aforementioned proposition that the same person can express different discounting patterns depending on the nature of the rewards.

### Recommendation for future research and practice

The adjustment of the rewards to the behavior postulated to be predicted by delayed rewards discounting should be implemented in further studies. When investigating precursors of financial behavior, the use of monetary rewards is likely to yield more accurate results. However, once the focus shifts to behavior with a potential for intrinsic rewards, then the type of rewards should be changed. Consequently, to address the inconsistencies related to the violent behavior, socially or intrinsically valued rewards should be given. Furthermore, such investigations into aggressive behavior should include covariates in the form of aggressiveness and hostility traits, as well as the potential to differentiate between reactive and proactive aggression.

Similarly, additional research is needed to verify the association between delayed rewards discounting and substance use, which could consider replacing monetary rewards. However, even with financial options, additional studies comparing forensic and general populations are required. Likewise, further investigation into psychopathy, specifically among adults who have offended, is necessary. Psychopathic traits potentially present in non-forensic populations are unlikely to exert the same effect on rewards choice as a cluster of entrenched symptoms found among forensic populations.

Nevertheless, from the perspective of maximizing utility, the primary aim of further research should be to identify interventions that can change the pattern of delayed rewards discounting among both forensic and the general population. Identifying them not only provides concrete tools that a person can use to alter their tendencies but also highlights the key mechanisms that affect the tendency to focus on short-term gains even when long-term ones outweigh them.

Although the current review could not find consistent evidence for delayed rewards discounting being a necessary or sufficient cause, it found support for treating such choice preference as a potential risk factor. Consequently, there are grounds to suggest the implementation of specific modules addressing preference for the small immediate rewards at the cost of larger but delayed ones. If such modules are implemented within existing treatments or interventions addressing cognitive structures supporting criminal conduct, then they can also address the potential problem of the forensic population not perceiving non-criminal life as a delayed but large rewards.

### Limitations

The current review is not without its limitations. Although all the included articles assessed delayed rewards discounting by presenting participants with two choices —one that grants a smaller gain in the short term and another that grants a larger gain in the long term —the particulars differed. Studies differed in the number of choices presented to the participants and the differences observed. This undermined the generalization of the results to the forensic population and could have been the source of the discrepancies in the results. Furthermore, despite the inclusion of female adults with criminal convictions in some of the studies, the majority focused exclusively on males, restricting the inference even further.

Another explicit limitation is the clustering of studies in countries with Western values, which prevents the investigation of the relationships between delayed rewards discounting and different cultures. However, the only study from a non-Western culture (Peng et al., 2022) still showed an increased tendency to discount large rewards if they are delayed, suggesting that this specific link *might* be cross-cultural. Lastly, as this study was a systematic literature review rather than a meta-analysis, the relationships highlighted in it are based on the qualitative analysis of the sources rather than quantitative indices.

### Conclusion

This review demonstrates that although delayed discounting of monetary rewards is associated with criminal conduct, the former can only be considered a risk factor, not a causal one. Not only does this relationship appear to be bidirectional, but the current evidence strongly supports it for crimes in general, but not for specific behaviors, such as violence. Nevertheless, the tendency to choose smaller immediate gains at the cost of larger ones in the future is not an entrenched algorithm applied to any two options. Instead, it is affected by the specific circumstances surrounding the choice as well as by the nature of the rewards and the individual’s cognitive structures. Thus, to accurately investigate its role in determining the behavior of adult offenders, individualized and conduct-relevant paradigms should be employed.

## Footnotes

1 In this article forensic population refers to those who have committed a criminal offence regardless of the presence of absence of a psychiatric diagnoses and the type of crime.

2 V = A / (1 – *k*D), where V is the perceived values of an outcome; A is an absolute value; D is delay; and k is the discount rate that determines the slope of the discounting function.
